# The complex relationship between MITF and the immune system: a Melanoma ImmunoTherapy (response) Factor?

**DOI:** 10.1186/s12943-020-01290-7

**Published:** 2020-12-05

**Authors:** Robert Ballotti, Yann Cheli, Corine Bertolotto

**Affiliations:** 1 Université Côte d’Azur, Nice, France; 2 Inserm, Biology and Pathologies of melanocytes, team1, Equipe labellisée Ligue 2020 and Equipe labellisée ARC 2019, Centre Méditerranéen de Médecine Moléculaire, Nice, France

## Abstract

The clinical benefit of immune checkpoint inhibitory therapy (ICT) in advanced melanomas is limited by primary and acquired resistance. The molecular determinants of the resistance have been extensively studied, but these discoveries have not yet been translated into therapeutic benefits. As such, a paradigm shift in melanoma treatment, to surmount the therapeutic impasses linked to the resistance, is an important ongoing challenge.

This review outlines the multifaceted interplay between microphthalmia-associated transcription factor (MITF), a major determinant of the biology of melanoma cells, and the immune system. In melanomas, MITF functions downstream oncogenic pathways and microenvironment stimuli that restrain the immune responses. We highlight how MITF, by controlling differentiation and genome integrity, may regulate melanoma-specific antigen expression by interfering with the endolysosomal pathway, KARS1, and antigen processing and presentation. MITF also modulates the expression of coinhibitory receptors, i.e., PD-L1 and HVEM, and the production of an inflammatory secretome, which directly affects the infiltration and/or activation of the immune cells.

Furthermore, MITF is also a key determinant of melanoma cell plasticity and tumor heterogeneity, which are undoubtedly one of the major hurdles for an effective immunotherapy. Finally, we briefly discuss the role of MITF in kidney cancer, where it also plays a key role, and in immune cells, establishing MITF as a central mediator in the regulation of immune responses in melanoma and other cancers.

We propose that a better understanding of MITF and immune system intersections could help in the tailoring of current ICT in melanomas and pave the way for clinical benefits and long-lasting responses.

## Treatments and resistance to treatments in melanoma

Cutaneous melanoma is a malignant tumor that develops from melanocytes and affects patients of all ages. The global incidence in 2015 was estimated to be 350,000, new cases [1] and is constantly increasing while the mortality is stable or in discrete increase [2]. The increased incidence is probably due to the improved early detection of thin forms, while the incidence of thicker forms which have the greatest impact on mortality has remained stable. Prior to 2011, no treatment has demonstrated a significant impact on the overall survival of patients with metastatic melanoma.

The first therapeutic revolution in the management of melanomas followed the discovery of activating mutations in *BRAF* (mainly *BRAF*^*V600E*^) in approximately 50% of melanomas in 2002 [3]. BRAF^V600E^ has also been identified in papillary thyroid cancer, non-small cell lung cancer, colorectal cancers and serous ovarian carcinoma. Vemurafenib and dabrafenib, specific BRAF^V600E^ inhibitors, that do not inhibit wild type BRAF, were the first targeted therapies used in metastatic melanoma which produced a spectacular response rate of 60% for the first time. However, secondary resistance develops within a few months, resulting in a poor increase in median progression-free survival (5 to 7 months) with BRAF^V600E^ inhibitor treatment [4]. A combination of BRAF^V600E^ and MEK inhibitors, which is now the standard of care, gives rise to a better response rate that reaches 75% [5] and delays the onset of resistance. Combination therapies therefore significantly prolonged median overall survival (up 25 months) and median progression-free survival (PFS) up to 12 months [6]. Despite this, resistance still occurs and worsens the clinical outcome of patients.

The second paradigm shift in melanoma treatment came from the use of monoclonal antibodies preventing the engagement of CTLA4 (Cytotoxic T-lymphocyte-Associated protein 4) or PD1 (Programmed Death protein 1), which are coinhibitory receptors expressed on the surface of T cells [7], with their ligands expressed on the surface of tumor cells. Anti-CTLA4 and anti-PD1 immune checkpoint inhibitor therapies (ICTs) prevent immune cell exhaustion and stimulate immune responses against tumor cells.

ICT has revolutionized the treatment of patients with cancer, to such an extent that Professors James Allison and Tasuko Honjo were awarded the 2018 Nobel Prize in Physiology or Medicine for their discovery of cancer therapy focused on inhibition of negative immune regulation. Adoptive T-cell therapy (ACT) can also induce durable antitumor responses due to the lasting memory feature of the adaptive immune system [8]. Interleukin 2 (IL-2), interferon, and oncolytic viral therapies can also have clinical benefits in melanoma, and they stand as alternatives for a subset of patients [9, 10].

ICTs have significantly improved outcomes for advanced-stage melanoma patients [11]. ICT agents show less impressive overall response rates (40% for anti-PD1 therapy and 20% for anti-CTLA4 therapy) than targeted therapies, while when combined they reach a response rate of more than 50% [12, 13]. More importantly, up to 40% of patients show a PFS after 4 years, indicating the development of long-lasting responses, and suggesting that this therapy may be a true cure. Nevertheless, these data also indicated that between 40 and 80% of patients display innate resistance to ICT and that when anti-CTLA4 and anti-PD1 antibodies are combined, at least 20% of patients develop secondary resistance. In addition, this drug combination can cause severe side effects.

There are multiple mechanisms of ICT resistance can be multiple, and their elucidation represents a challenge that researchers, clinicians and the pharmaceutical industry are facing now.

A complete deciphering of these mechanisms will allow the development of rationalized combinatorial treatments and further improve patients’ overall survival.

A substantial amount of work has led to the identification of the mechanisms of resistance to ICT in melanomas and, more generally, solid cancers (for review see [14, 15]. In brief, resistance can be ascribed to (i) a poor immunogenicity of the tumor, due to defective antigen expression or presentation in a context impairing immune cell activation, (ii) an altered T-cell trafficking to the tumor and (iii) reduced T-cell killing activity within the tumor. All these effects are mediated mainly by the tumor itself, but also by the tumor microenvironment (inflammation, hypoxia composition, matrix make-up, nutrient availability, etc.), composition of the immune infiltrate and by long-distance signaling arising from the gut microbiota.

## Pivotal role of MITF in the response of melanoma to immunotherapies

Here, we focus our attention on intrinsic and melanoma specific mechanisms that could account for ICT therapy resistance and that would be amenable targets for new therapeutic strategies. Melanoma-specific mechanisms almost universally involve the MIcrophthalmia-associated Transcription Factor (MITF). MITF comprises eight isoforms differentially expressed within various cell types and tissues [16]. The MITF-M isoform (hereafter simply designated as MITF) is the master regulator of melanocytes and has been identified as an addictive oncogene in melanoma [17–20].

The leading hypothesis in the field is that high MITF expression (MITF^high^) is associated with a differentiated and proliferative phenotype, whereas low MITF expression (MITF^low^), is associated with a dedifferentiated, invasive, apoptosis-resistant, and melanoma-initiating cell phenotype [21]. MITF^high^ and MITF^low^ cells coexist in melanoma tumors [22–24], and can originate from a reversible phenotypic switch that is responsible for melanoma plasticity and intratumor heterogeneity [25]. Signals in the tumor microenvironmental, such as hypoxia, nutrient availability and cytokines, that dampen MITF levels, can also favor the phenotypic transition [23, 26–29]. Importantly, MITF has been shown to be instrumental in the response to targeted therapies, yet its role is complex given that both increased and decreased MITF expression can mediate resistance to BRAF inhibitors [24, 30, 31]. In addition, increasing evidence has been gathered, suggesting a role of MITF in the resistance to immunotherapy. This role in the resistance to immunotherapies is more ambiguous than in resistance to targeted therapies and has not yet been completely integrated by the community from therapeutic perspectives aimed at combatting resistance.

In this review, we focused our attention on MITF functions related to immunity, that could account for ICT resistance.

## Role of MITF in antigen expression and presentation

Antigens are recognized by T cells when they are displayed on cell surfaces by Major Histocompatibility Complex (MHC) molecules. Antigen presentation is carried out by antigen-presenting cells (APCs), the most important of which are dendritic cells (DCs), B cells and macrophages. Notably, some tumor cells including melanoma cells, can also be considered as APCs. MHC expression is controlled by interferon gamma (IFNγ), which is primarily produced by cells of the immune system and binds to the heterodimeric receptor complex, IFNGR1/IFNGR2, on tumor cells, activating the JAK1/2-STAT1 signaling cascade [32]. Inactivating mutations in the antigen presentation pathway such as mutations in beta 2 microglobulin (β2M) and JAK1/2 can influence the ability of melanoma cells to present peptides to the immune system. Loss-of-function mutations in *JAK1*/*2* can lead to both primary and acquired resistance to anti-PD1 therapy [33, 34]. *JAK* mutations in tumor cells impair signaling initiated by IFN-γ, leading to acquired resistance to anti-PD1 therapy. Likewise, in primary resistance to anti-CTLA4 therapy, a relatively high frequency of mutations in several molecules involved in the interferon signaling pathway was reported [35].

If reduced MHC expression precludes efficient tumor cell recognition by T-cells, lack iofn antigen presentation is also a limiting step.

Indeed, MITF functions as a central regulator of melanocyte differentiation and melanogenesis by controlling the transcription of a panel of genes, including melan-A (MART1), tyrosinase and premelanosome protein PMEL17 (PMEL/SILV/gp100) [36–38], which encode proteins that have been reported to be tumor-specific antigens [39, 40].

**Fig. 1:**
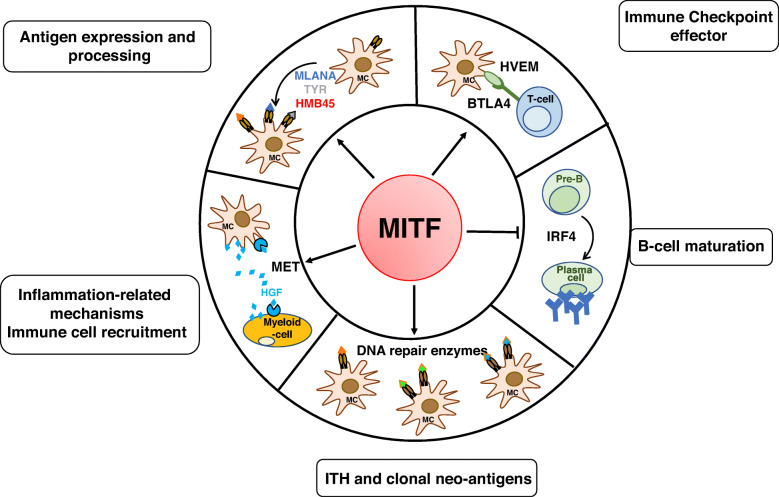
Summary schematic of the hallmarks of MITF intersection with the immune system at the transcriptional level. MITF, which is associated with melanoma cell differentiation, controls antigen expression and processing. By regulating the expression of DNA repair enzymes, MITF might be involved in neoantigen formation. MITF also reduces inflammatory molecule secretion and tumor heterogeneity, resulting in enhanced T cell recognition and immunosurveillance. By contrast, MITF, through HVEM, which prevents an efficient immune response, through MET upregulation which attracts neutrophils, and through reduced expression of IRF4 which dampens B cell maturation, may favor the immunosuppression. BTLA, B- and T-Lymphocyte Attenuator; CCL, Chemokine (C-C motif) ligand; HGF, Hepatocyte Growth Factor; IFN, Interferon; IL, interleukin; IRF, interferon regulatory factor; MC, melanocyte; TNF-α, tumor necrosis factor alpha; TYR, tyrosinase

Antigenic peptides are derived from proteins degraded by acidic proteases in the endolysosomal pathway. In melanomas, this function can also be assumed by specialized, lysosome-related organelles called melanosomes [40, 41]. Knowing that MITF drives endolysosomal and melanosomal biogenesis and functioning [42], MITF can affect antigen processing in addition to antigen production (Fig. 1).

Supporting this idea, melanosomes and endolysosomes harbor distinct processing abilities that modify the presentation of MHC-restricted epitopes [40]. Since melanomas can lose their differentiated phenotype, in part due to downregulation of MITF expression, and subsequently no longer contain identifiable melanosomes, this change could affect antigen processing and presentation and influence immune recognition.

Consequently, any reduction in MITF expression should affect surface antigen presentation and recognition of melanoma cell recognition by the immune system.

## Roles of MITF in the mutational status and intratumor heterogeneity

Tumor cells are genetically unstable, displaying the accumulation of somatic mutations in their genome, which is believed to increase the likelihood of immunogenic neoantigens expression by tumor cells and to favor T cell recognition. The high tumor mutational burden (TMB) in melanoma results from exposure to ultraviolet radiation (UV) radiation [43–45]. The TMB has emerged as a clinically relevant biomarker of ICT efficacy [46–49]. The higher the mutational burden is, the better the response to immune checkpoint inhibitors [50]. Patients with tumors, including cutaneous melanomas, harboring dysfunctional in DNA repair mechanisms, are better responders to anti-PD1 therapy than those with intact DNA repair mechanisms [51, 52]. A striking example is patients with uveal melanoma another type of melanoma, that is resistant to all immunotherapy regimens, however, a subset of patients, harboring inactivating mutations in methyl-CpG-binding domain protein 4 (MBD4) exhibit sensitivity to ICT [53, 54]. MBD4 is involved in base excision repair, and those responding uveal melanomas are characterized by a high mutational burden. *Mbd4*-deficient mice, which display an enhanced mutation frequency, can be used to generate more physiopathological information on the roles of MBD4 in tumorigenesis and response to immune function [55–57]. Remarkably, MITF safeguards genomic stability through the transcriptional regulation of distinct DNA repair genes such as FANCA, BRCA1, POLE4 and POLD4, and MLH1 [37, 58–60], the last being a binding partner of MBD4 [61]. It is somewhat intuitive that the the phenotypic transition towards a MITF^low^ state should therefore favor genomic instability, an increase in the umor mutational burden and the likelihood of neoantigen formation, rendering the cells more immunogenic. This is in contrast with data from the literature indicating that MITF^low^ cells are more resistant to immunotherapies. This is likely because, MITF^low^ cells switch towards a dedifferentiated phenotype and harbor low expression of immunogenic target antigens, rendering them “poorly visible” for T cells. In addition, MITF^low^ cells produce a pro-inflammatory secretome, that could ultimately affect T-cell recruitment and function (Fig. 1).

However, a high mutational load alone appears to be insufficient for producing clinical benefit. Indeed, some tumors showing a high mutational load do not respond to immunotherapy [62], whereas, tumors with a low mutational burden can respond well [63]. Importantly, not all neo-antigens are responsible for the formation of efficient CD8+ clones. In addition to accumulation of mutations and the generation of immunogenic neopeptides, genomic instability also allows the emergence of new less immunogenic new clones, that escape immune surveillance, and favor primary or acquired resistance. In line with that, UVB, the most common environmental risk factor for melanoma, enhances the neoantigen burdens, favoring melanoma aggressiveness [64]. Given that MITF has roles in DNA repair, ITH and the expression of differentiation antigens, a comprehensive understanding of its role in these processes is a major asset to better understand melanoma resistance to immunotherapies.

## Interactions of MITF and the pathways affecting the immune response of melanoma

Specific oncogenic signals have been shown to mediate cancer immune evasion and resistance to immunotherapies, highlighting new putative targets for immune potentiation/rescue.

BRAF^V600E^, the most frequent mutation in cutaneous melanomas, downregulates the expression of MITF and its downstream effectors operating in the differentiation pathway, while MITF expression is increased by BRAF inhibitors [65, 66]. Given that MITF can impact on antigen presentation, this holds promise for the combination of targeted therapies with ICT. In humans, a the clinical trial (NCT02130466) revealed that triple-combination therapy (dabrafenib+trametinib+anti-PD1 antibody) increases the frequency of long-lasting antitumor responses in a subset of patients with BRAF^V600^-mutated metastatic melanomas [67]. Notably, in a preclinical model, a quadruple-combination therapy comprising dabrafenib, trametinib, an anti-PD-1 or anti-PD-L1 antibody and an immunostimulatory antibody specific for CD137 or anti-CD134 resulted in an effect superior to that of te triple-combination therapy [68].

**Fig. 2:**
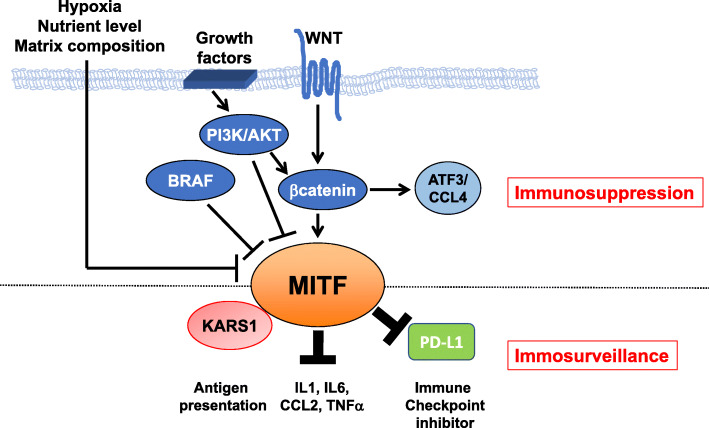
Summary schematic of MITF regulation by oncogenic pathways and the intersection of MITF with the immune system at the post-translational level. Oncogenic signaling pathways (WNT/β-catenin, PI3K/AKT and BRAF) in melanoma cells as well as the tumor microenvironment signals all converge to regulate MITF levels and/or activity and immune function, favoring immunosuppression. By interacting with KARS1, repressing PD-L1 expression and reducing cytokine secretion MITF favors immunosurveillance

Of interest, a screen of natural molecules related to aloperine led to the identification of SA-49, which induces a MITF dependent degradation of PD-L1 through the lysosomal pathway [69]. Therefore, targeted therapies, through increased MITF expression, may in turn decrease PD-L1 expression and improve the immune response and ICT efficacy (Fig. 2).

Hepatocyte growth factor (HGF), via stimulation of its cognate receptor c-MET, and activation of the downstream effector components (MAPK, STAT, PI3K-AKT cascades and NF-κB), increases the survival, motility and proliferation of several cell types of cancer cells, including melanoma cells. The role of HGF/MET signaling in the immune system in cancer is gaining attention since it could constitute a mechanism of primary and acquired resistance to cancer immunotherapy [70]. Autologous T cells expressing cMET CAR mRNA are being evaluated in patients with advanced melanoma or breast carcinoma in clinical trials (NCT03060356). Recently, some approaches have also been developed to combine MET and PD1/PD-L1 inhibition in locally advanced or metastatic hepatocarcinoma and renal cell carcinoma (NCT03655613 and NCT03672305). HGF can be produced by stromal cells and act on neutrophil recruitment [71] to limit therapeutic efficacy. High neutrophil-to-lymphocyte ratios in patients with advanced cancer including melanoma generally correlate with a poor response to ICT and a poor prognosis [72–74]. HGF can also be secreted by the cancer cells themselves and exert its effect in an autonomous manner to regulate the expression of its own receptor in a MITF-dependent manner [75, 76]. Upregulation of MET expression on tumor cells leads to an exacerbated HGF signaling and allows HGF to protect melanoma cells from apoptosis [75], highlighting a feed forward loop between HGF and cMET to favor treatment resistance (Fig. 1).

The PI3K/AKT pathway is also frequently activated in melanoma cells. One of the most common activation mechanisms is by loss of function of the tumor suppressor PTEN. PTEN loss often occurs in conjunction with BRAF mutation and is associated with relatively poor outcomes [77, 78]. PTEN-deficient melanoma cells tend to be less immunogenic than wild-type melanoma cells and are resistant to T cell-mediated immunotherapy [79]. One possible explanation is that the PI3K pathway, through increased BRN2 expression, dampens the level of MITF, and consequently inhibits the differentiated melanocytic phenotype [65, 80, 81]. Furthermore, PTEN deficient melanoma cells produce inhibitory cytokines such CCL2 and VEGF, that impede immune infiltration and T cell function. Interestingly, PI3K inhibitors and especially inhibitors of the PI3Kβ isoform, could represent a new weapon against melanoma since they have been shown to improve the activity of both anti-PD1 antibodies and anti-CTLA4 antibodies in murine models [79]**.** Notably, PI3K inhibition has been reported to upregulate the expression of MITF level and differentiation antigens [82] (Fig. 2).

Downstream of PI3K, WNT/beta-catenin (β-catenin) is another important signaling pathway that is implicated in many cancers including melanoma and which has been shown to play a key role in immune response [83–85]. Tumor-intrinsic active β-catenin signaling, ensuing gain-of-function mutations in β-catenin, loss-of-function mutations in negative regulators of WNT signaling and increased expression of β-catenin effectors, result in T cell exclusion and “cold tumor” phenotype. The subset of melanomas with active β-catenin signaling shows resistance to ICT [86–88]. Trujillo et al., also have demonstrated that secondary resistance to immunotherapies might arise upon selection for strong tumor expression of β-catenin mediating tumor T cell exclusion from the tumor [87]. The combination of WNT/β-catenin signaling inhibitors with ICT may be a valid therapeutic option. Of interest, β-catenin regulates the transcription and expression of MITF in the melanocyte lineage and melanoma cells, potentially driving their differentiation or growth [27, 89–91]. Mechanistically, β-catenin can interact with Mitf in a Lef1-dependent [92], or Lef1-independent [93] manner. It has been hypothesized that Mitf can redirect β-catenin transcriptional activity away from β-catenin/Lef1-regulated genes towards Mitf-specific target promoters, thereby diversifying the cellular behaviors. Remarkably, β-catenin signaling has also been reported to increase BRN2 expression via LEF-binding sites [94], and BRN2 represses MITF transcription [65, 80, 81]. Thus, β-catenin inhibition could switch the MITF level from high to low favoring dedifferentiation, and reduced immunogenicity (Fig. 2). Thus, while therapeutic options aimed at targeting WNT/β-catenin signaling may hold promise to turn cold tumors into hot ones, they need to be considered with caution owing to a potential effects of reduced antigen presentation and the induction of an inflammatory TME induction due to MITF inhibition.

Mitf can also acts as a transcriptional repressor of genes involved in type I innate immune signaling in mouse melanocytes [95]. Although MITF binds and activates interferon regulator factor 4 (Irf4) in human melanocytes [96], and can transcriptionally regulate IFN signaling [97], regulation of the innate immune genes does not seem to involve Irf4. Upregulation of the expression of a fraction of these innate immune genes has also been observed in human melanoma cells treated with shRNA specific for MITF [98]. These findings strongly suggest a role for MITF in regulating the innate immune responses.

Immune responses are regulated by the engagement of costimulatory and coinhibitory receptor molecules. Immunotherapeutics targeting the inhibitory receptors CTLA-4, PD-1 or PD-L1 have achieved substantial clinical progress in cancer. However, a large proportion of patients remain unresponsive to these treatments, which may be due to the expression of other coinhibitory receptors on the surface of tumor infiltrating lymphocytes rendering anti-CTLA4 or anti-PD-1 monotherapy ineffective. Coinhibitory receptor targets that are being explored in clinical trials include LAG-3, TIM-3, TIGIT and HVEM [99, 100]. Some of these coreceptors have already been targeted in clinical trials (ClinicalTrials.gov). Specifically, Herpes Virus Entry Mediator (HVEM, also known as TNFRSF14), a member of the TNF receptor superfamily and thought to be primarily expressed on hematopoietic cells, has also been described to be expressed on melanoma cells [101]. Metastatic melanoma patients, with a high HVEM expression, have a significantly poorer overall survival than those with low HVEM expression [102]. HVEM on melanoma cells inhibits the proliferation and production of IFNγ by B- and T-lymphocyte attenuator (BTLA)-positive tumor-specific CD8 T-cells. BTLA is an immunoregulatory receptor, similar to CTLA-4 and PD-1, and is mainly expressed on B cells, T cells and all mature lymphocytes [103]. These results suggest the transduction of inhibitory signals caused by HVEM/BTLA interactions. Importantly, HVEM was recently reported to be a target gene of MITF [102], indicating that MITF also regulates antimelanoma immune responses at the level of the regulatory function of coinhibitory receptors. In this case, MITF would favor immune cell exhaustion. Thus, anti-BTLA therapy warrants investigation in patients resistant to anti-CTLA4 or anti-PD1 therapy (Fig. 1).

## The tumor microenvironment

There is growing evidence that the production of cytokines or other factors in the tumor microenvironment decreases T-cell expansion and function and may help melanoma cells to resist elimination by T lymphocytes.

For instance, the immune cell-rich microenvironment driven by tumor-initiated inflammation promotes melanoma dedifferentiation and overrides protective adaptive immunity [104, 105]. Consistently, a previous study indicated that an inflammatory milieu induces melanoma dedifferentiation, illustrated by a reduction in the levels of melanocytic antigens, and favors immunotherapy resistance [106].

Mechanistically, T cell-driven inflammatory stimuli such as TNF-α, engage the transcription factor c-jun in tumor cells, which in turn reduces MITF levels, and consequently decreases the expression of melanocyte differentiation genes, thereby leading to melanoma cell dedifferentiation [107]. This is reminiscent of a previous report demonstrating that TNF-α, via activation of NFκB, reduces the expression of melanocyte differentiation genes, likely through decreased MITF expression [108]. These observations support the idea of intervening with anti-TNF-α antibodies. Consistent with this, nivolumab+ipilimumab in combination with the anti-TNF-α antibody certolizumab has been studied in advanced melanoma in clinical trial (NCT03293784) [109].

Other cytokines such as IFN-γ, IL-1β, IL-6 and CCL2, produced by the TME also induce dedifferentiation and phenotypic transition as well. IFN-γ, which is released by T cells and tumor-infiltrating myeloid immune cells, also diminishes the mRNA levels of melanocyte differentiation antigens [110]. Further work is required to determine how IFN-γ functions to affect melanocyte differentiation antigens, this process might involve the downregulation of MITF expression [111]. Likewise, IL-1β strongly reduces the expression of MITF and melanocyte differentiation antigens. This translates into a reduced recognition of melanoma cells by cytolytic T lymphocytes [112]. Mechanistically, IL-1β inhibits MITF through microRNA-155 [113].

It is well known that some of these factors can also be secreted by melanoma cells themselves. MITF may be involved in this production. Indeed, compared to MITF-positive cells, the MITF-negative cells produce larger amounts of IL-1α and IL-1β. Furthermore, the supernatant of MITF-negative melanoma cells reduces MITF expression in positive cells. This effect is blocked by the IL-1 receptor antagonist IL-1Ra [112].

In agreement with the above information, genetic suppression of MITF in melanoma cells (siRNA-mediated MITF knockdown) triggers an inflammatory secretome comprising the abovementioned cytokines [28, 60, 114]. Naive melanoma cells exposed to the inflammatory secretome of MITF-depleted cells switch towards a dedifferentiated phenotype with a reduced immunogenicity ability that could contribute to escape from immune responses. This phenotype can partially be rescued by anti-CCL2 neutralizing antibodies [114]. MITF inhibits the promoter activity of CCL2, suggesting that MITF controls the expression of a CCL2 repressor [114]. Likewise, IL6 via activation of the JAK/STAT3 signaling pathway induces a sharp decrease in the levels of MITF and its upstream regulator PAX3, both of which are associated with dedifferentiation [115]. Collectively, these observations implicate MITF in the regulation of cytokine production and suggest the existence of a negative-feedback loop between MITF, IL1 and the CCL2 family of cytokines. Oncogenic BRAF, through decreased MITF expression and enhanced IL-1α and IL-1β secretion, triggers PD-L1 and PD-L2 expression in tumor-associated fibroblasts which also contributes to suppression of tumor-infiltrating T cell function [116].

Notably, that there is greater immune infiltration in MITF^low^ tumors than in MITF^high^ tumors. This is likely because MITF regulates the key lipogenic enzyme stearoyl-CoA desaturase (SCD) [27]. Low SCD levels leads to inflammatory signaling and immune cell recruitment, although dedifferentiated MITF^low^ cells are not responsive to immune function. Thus, enhancing the MITF-SCD axis which suppresses inflammatory signaling, may represent a therapeutic strategy to improve ICT outcomes.

Variations in other microenvironmental cues, including hypoxia, matrix composition, nutrient levels and the microbiota, can also influence the therapeutic response to ICT. Crucially, the transcriptomic profiles of anti-PD1 monotherapy nonresponders exhibit upregulation of the expression of the hypoxic marker, carbonic anhydrase 9 [117], which is a known cancer progression marker [118]. In line with this, hypoxia reduces MITF levels [23, 119], an effect in part mediated through the transcriptional repressor bHLHb2 [23].

Tumor metabolism shapes antitumor immune responses [120]. Several lines of evidence indicate that metabolic changes regulate MITF levels and thus may impact on melanoma immunogenicity [26, 27, 29, 121]. Melanoma cells can also express the enzyme indoleamine 2,3-dioxygenase (IDO) in response to IFN-γ. IDO contributes to immunosuppression by catalyzing the degradation of tryptophan into kynurenine which causes an inhibition of effector T cell functions [122]. Notably, the kynurenine pathway is essential for the de novo synthesis of NAD^+^. Importantly, increased NAD+ levels render BRAF^V600E^ melanoma cells resistant to vemurafenib and this was associated with epigenetic changes and reduced MITF expression [121]. One can thus envision that increased NAD+ levels, favors MITF downregulation, thereby supporting ICT resistance.

In conclusion, MITF loss drives melanoma cell plasticity and phenotypic switching that correlates with reduced differentiation and immunogenicity, this loss also limits antitumor efficacy through the production of an inflammatory milieu that shapes an immunosuppressive TME.

Finally, several lines of evidence also support the key role of the gut microbiota in the control of tumor growth and response to therapy [123–126]. The gut microbiome composition could predict treatment response and may contribute to immune responses. Thus, modulating the gut microbiome in patients receiving ICT (antibiotics, prebiotics, or bacterial introduction, or of bacterial metabolic byproducts such as short-chain fatty acids or conjugated bile acids) offers a new therapeutic strategy in patients with primary resistance and could nicely complement established treatments for melanoma. Whether MITF can impact on the microbiota composition or vice versa has yet to be demonstrated.

## Roles of MITF in nonmelanoma cells

As mentioned earlier, whereas the M-isoform of MITF is specifically expressed in the melanocyte lineage, MITF comprises other isoforms expressed within various cell types.

The *MITF* E318K germline mutation predisposes to melanoma, but also renal cell carcinoma (RCC) [17]. A rare subtype of RCC, translocation renal cell carcinoma (tRCC), which occurs in patients before 40 years, harbors specific translocations of *TFE3*, *TFEB* and MITF leading to their overexpression [127]. tRCC displays relatively aggressive behavior and appears resistant to immune checkpoint inhibitors after a first-line treatment with tyrosine kinase inhibitors [128]. Hence, in the context of RCC, high MITF levels appear to be associated with ICT resistance.

Dendritic cells, the most potent APCs, express MITF. An interaction between MITF and the lysyl-tRNA synthetase (KARS1) has been reported to stimulate MITF transcriptional activity through the generation of diadenosine tetraphosphate (Ap4A), which is also a key regulator of antigen presentation [129]. Thus, MITF can influence the initiation and maintenance of primary immune responses. Furthermore, mast cells also play an important role in both innate and adaptive immunity [130, 131]. Interestingly, MITF is required for their proper development and function, notably through regulating KIT expression [132]. It is worth noting that the regulation of KIT by MITF in melanocytes and melanoma cells remains to be clearly demonstrated, indicating that a tissue specific regulation of KIT by MITF in mast cells might be explained by expression of lineage-restricted cofactors.

Furthermore, in naive B cells, MITF represses IRF4, a critical regulator of various aspects of B- and T cell maturation [133, 134]. In mice, Mitf antagonizes the process of B cell terminal differentiation into antibody secreting plasma cells. Conversely, defective Mitf activity results in spontaneous B cell activation and antibody production [135]. Thus, the MITF level or MITF activity can also affect T and B cell maturation.

Interestingly, MITF has been reported to play a key role in immune defense in the mollusc clam, *Meretrix* petechialis. Although not related to the cancer field, this model can provide robust insights into how MITF functions in immunity [136].

## Conclusion

Although it is clear that MITF has a role in immune cell trafficking and function, its roles are complex and, as it is often the case with MITF, is marked by a certain duality. Indeed, MITF loss was expected to reduce differentiation and antigen expression, thereby decreasing immunogenicity. Immunogenicity can be restored by reintroducing differentiation antigens as it has been done by using nanoparticulate liposomal RNA vaccine encoding tumor-associated antigens such as tyrosinase. This strategy has recently demonstrated a durable objective response in ICT-treated patients with advanced melanomas [137]. Furthermore, given that MITF^low^ melanoma cells produce an inflammatory secretome [114], identification and targeting of factors and/or chemokines in this secretome, preventing antitumour immunity, could also improve response to ICT. In line with this, transient MITF knockdown, leads to decreased immune cell recruitment in B16 melanoma tumors [138]. However, in the TCGA cohort, MITF^low^ tumors displayed an increased immune cell infiltrate that could be explained by a consequent downregulation of SCD expression [27]. Whether this immune infiltrate has a fully functional cytotoxic function remains to be studied.

Also of interest is the peculiar link between MITF and KARS1, which operates in melanocytes and other cell types such as DCs [139–141] (Fig. 2).

The most striking links between MITF and the immune responses were identified in a recent study using four syngeneic models recapitulating diverse subtypes of human melanoma and the diversity of clinical responses to ICT. This study pointed out to a melanocytic plasticity signature predictive of patient outcomes in response to immune checkpoint blockade [142]. The study results also suggest that a high differentiation status in melanoma predicts ICT benefit. In particular, the expression of several MITF target genes was upregulated in the differentiation signature, however, that of MITF, whose activity can be regulated by posttranslational events, such as those mediated by KARS1, rather than at the transcriptional level, was not. In agreement with these observations, analysis of the transcriptomic signature of melanoma patients according to their response to anti-PD1 therapy also identified a dedifferentiation signature (downregulation of MITF target gene expression) associated with a lack of response to the treatment [143].

Therefore, accumulating evidence indicates that MITF has an important impact on immune function and the response to ICT, acting both on tumor immunogenicity and in shaping the immunological TME. Although, MITF favors melanoma proliferation, it might be worth evaluating the effects of agents that increase MITF expression on the response to ICT. Such agents include alpha-melanocyte-stimulating hormone (α-MSH) and cAMP-elevating agents, such as forskolin that were reported to increase the levels of MITF and melanoma antigens such as MART-1 and GP-100 [144, 145]. These treatments are expected to increase the recognition of melanoma cells by T cells and improve the immune response. Similar results may be obtained with methotrexate [146]. In summary, one of the main hurdles to achieving fully effective immunotherapy is the plasticity of melanoma cells, in which MITF plays a pivotal role. This plasticity makes of melanoma a moving target that is difficult to target with the immune system. Therefore, freezing melanoma cells in one phenotypic state, preferably the differentiation state, may be of interest to facilitate antitumor effects by the immune system.

Finally, it may be reasonable to develop CAR-T cells targeting proteins highly expressed in dedifferentiated melanoma, such as AXL, whose expression is clearly inversely correlated with that of MITF.

In conclusion, it may be wise to include MITF in the pipeline of reflection aimed at finding rationalized and personalized treatments, that combine multimodal therapeutic approaches with ICT to prevent resistance relapse and generate long-term survival benefits.

## Data Availability

Not applicable.

## Abbreviations

ACT: Adoptive T-cell Therapy
APC: Antigen-presenting cells
AKT (PKB): Protein Kinase B
β2M: beta 2 microglobulin
bHLHB2: basic Helix Loop Helix protein class B2
BRAF: B-Raf Proto-Oncogene
BRCA1: BReast CAncer 1
BTLA: B- and T-Lymphocyte Attenuator
CCL2: Chemokine Ligand 2
CTLA-4: Cytotoxic T-Lymphocyte-Associated protein 4
DC: Dendritic Cells
FANCA: FANConi anemia group A protein
HGF: Hepatocyte Growth Factor
HVEM: Herpes Virus Entry Mediator
ICT: Immune Checkpoint inhibitory Therapy
IL: Interleukin
IFN: Interferon
IFNGR: Interferon gamma receptor
ITH: IntraTumor Heterogeneity
IRF4: Interferon Regulatory Factor 4
JAK: Janus Kinase
KARS: Lysine-tRNA ligase
LAG3: Lymphocyte-Activation Gene 3
LEF1: Lymphoid Enhancer Binding Factor 1
NAD: Nicotinamide adenine dinucleotide
MAPK: Mitogen Activated Protein Kinase
MART1: melanoma antigen recognized by T cells 1
MHC: Major Histocompatibility Complex
MBD4: Methyl-CpG Binding Domain 4
MITF: MIcrophthalmia-associated Transcription Factor
MLH1: MutL Homolog 1
NFκB: Nuclear Factor kappa B
PD1: Programmed Death protein 1
PD-L1: Programmed Death-Ligand 1
POLD4: Polymerase (DNA-directed) delta 4
POLE4: Polymerase (DNA-directed) epsilon 4
PMEL17: PreMELanosome protein
PTEN: Phosphatase and TENsin homolog
RCC: Renal Cell Carcinoma
SCD: Stearoyl-CoA Desaturase
STAT: Signal Transducer and Activator of Transcription
TCGA: The Cancer Genome Atlas
TFEB: Transcription Factor EB
TFE3: Transcription Factor E3
TIGIT: T cell immunoreceptor with IG and ITIM domains
TIM-3: T-cell Immunoglobulin and Mucin-domain containing-3
TNF: Tumor Necrosis Factor
TME: Tumor microenvironment
TMB: Tumor Mutational Burden
UV: Ultraviolet
WNT: Wingless
